# Diversity, genetic mapping, and signatures of domestication in the carrot (*Daucus carota* L.) genome, as revealed by Diversity Arrays Technology (DArT) markers

**DOI:** 10.1007/s11032-013-9979-9

**Published:** 2013-10-26

**Authors:** Dariusz Grzebelus, Massimo Iorizzo, Douglas Senalik, Shelby Ellison, Pablo Cavagnaro, Alicja Macko-Podgorni, Kasia Heller-Uszynska, Andrzej Kilian, Thomas Nothnagel, Charlotte Allender, Philipp W. Simon, Rafal Baranski

**Affiliations:** 1 Insitute of Plant Biology and Biotechnology, University of Agriculture in Krakow, Al. 29 Listopada 54, 31-425 Krakow, Poland; 2 Department of Horticulture, University of Wisconsin-Madison, 1575 Linden Drive, Madison, WI 53706 USA; 3 Vegetable Crops Research Unit, USDA-Agricultural Research Service, University of Wisconsin, 1575 Linden Drive, Madison, WI 53706 USA; 4 CONICET and INTA EEA La Consulta, CC8 La Consulta (5567), Mendoza, Argentina; 5 Diversity Arrays Technology Pty Ltd, 1 Wilf Crane Crescent, Yarralumla, ACT 2600 Australia; 6 Institute for Breeding Research on Horticultural and Fruit Crops, Julius Kühn-Institut, Federal Research Centre for Cultivated Plants, Erwin-Baur-Str. 27, 06484 Quedlinburg, Germany; 7 Warwick Crop Centre, University of Warwick, Wellesbourne, Warwick, CV35 9EF UK

**Keywords:** Carrot, DArT, Domestication, Diversity structure, Linkage mapping, Selection

## Abstract

Carrot is one of the most economically important vegetables worldwide, but genetic and genomic resources supporting carrot breeding remain limited. We developed a Diversity Arrays Technology (DArT) platform for wild and cultivated carrot and used it to investigate genetic diversity and to develop a saturated genetic linkage map of carrot. We analyzed a set of 900 DArT markers in a collection of plant materials comprising 94 cultivated and 65 wild carrot accessions. The accessions were attributed to three separate groups: wild, Eastern cultivated and Western cultivated. Twenty-seven markers showing signatures for selection were identified. They showed a directional shift in frequency from the wild to the cultivated, likely reflecting diversifying selection imposed in the course of domestication. A genetic linkage map constructed using 188 F2 plants comprised 431 markers with an average distance of 1.1 cM, divided into nine linkage groups. Using previously anchored single nucleotide polymorphisms, the linkage groups were physically attributed to the nine carrot chromosomes. A cluster of markers mapping to chromosome 8 showed significant segregation distortion. Two of the 27 DArT markers with signatures for selection were segregating in the mapping population and were localized on chromosomes 2 and 6. Chromosome 2 was previously shown to carry the *Vrn1* gene governing the biennial growth habit essential for cultivated carrot. The results reported here provide background for further research on the history of carrot domestication and identify genomic regions potentially important for modern carrot breeding.

**Electronic supplementary material:**

The online version of this article (doi:10.1007/s11032-013-9979-9) contains supplementary material, which is available to authorized users.

## Introduction

Carrot is one of the most important root vegetable crops grown worldwide, on more than one million hectares (FAOSTAT 2011). Its progenitor, wild *Daucus carota* L., is a weed commonly occurring across continents in the temperate climatic zone. However, carrot has not been studied extensively and molecular tools facilitating genome analysis and breeding of the crop remain underdeveloped. Only recently has a more systematic approach towards developing such tools been carried out, resulting in a set of simple sequence repeat (SSR) and single nucleotide polymorphism (SNP) markers identified through sequencing of carrot bacterial artificial chromosome (BAC) ends (Cavagnaro et al. 2009) and comparative analysis of three carrot transcriptomes (Iorizzo et al. 2011). Both marker systems were subsequently used for analysis of genetic diversity of *D. carota*, corroborating an earlier hypothesis on carrot evolution formulated on the basis of morphological observations by Small (1978). Iorizzo et al. (2013a, b) provided clear evidence for diversification between wild and cultivated accessions, supporting previous reports based on amplified fragment length polymorphism markers (Shim and Jørgensen 2000; Bradeen et al. 2002). Analysis of SSR markers by Baranski et al. (2012a) showed evidence for the separation of the cultivated germplasm into two distinct groups, the Eastern (Asian) and Western (European and American) gene pools. A recent study based on *D. carota* plants of different origin genotyped with more than 3,300 SNP markers suggested that Central Asia is the center of origin of cultivated carrot, and that orange-rooted carrots of the Western type were selected from the yellow domesticated carrots (Iorizzo et al. 2013a). Traits important for primary domestication included the ability to form fleshy roots, minimal lateral root branching and biennial growth habit, while further improvement required selection for a range of traits determining root quality, e.g. shape, color, smoothness etc. Generally, Western carrots appear as a more advanced group, better adapted for commercial production and processing. They usually develop roots of cylindrical or tapered cylindrical shape favored by the food processing industry, have less pubescent leaves and show little tendency for bolting (Rubatzky et al. 1999). They are also characterized by high content of pro-vitamin A carotenoids, mostly β-carotene responsible for the orange root color (Simon 2000). Additionally, Western carrots are sweeter, having on average 18 % higher sugar content than Eastern carrots (Baranski et al. 2012b). Eastern carrots commonly grown in Asia produce rather thicker, shorter roots or narrow conical roots with a tendency for branching in some varieties. They often have pubescent leaves and tend to flower early, and hence they exhibit more primitive traits (Rubatzky et al. 1999). Their roots are poor in provitamin A carotenoids and have yellow (lutein), purple (anthocyanin) or red (lycopene) color. In contrast to Western carrots, they are richer in phenolic compounds, resulting in higher radical scavenging activity of the root extract that is particularly well exhibited in purple roots containing anthocyanins (Leja et al. 2013). However, despite the apparently high selective pressure imposed on the domesticated population, Iorizzo et al. (2013a) observed no reduction in genetic diversity resulting from a domestication-related bottleneck in Eastern and Western gene pools of cultivated carrot.

Here, we attempted to find out whether, despite the high overall level of genetic diversity, any particular regions showing shifts in allele frequency that could be attributed to the domestication process were present in the carrot genome. To achieve our goal, we used a Diversity Arrays Technology (DArT) platform—a microarray-based molecular marker system allowing cost-efficient (per data point) high-throughput genotyping of any organism. Importantly, DArT genotyping does not require any prior knowledge of the genome sequence (Jaccoud et al. 2001). It has been widely applied in plant science and proven to perform well for many species (Kilian et al. 2005). It provides means for simultaneous genotyping of hundreds to thousands of dominant DArT markers resulting from nucleotide polymorphisms within restriction enzyme recognition sites. We report on the development of the DArT platform for carrot genotyping, based on genomic representations derived from a carrot diversity collection, including wild and cultivated accessions. The platform was used to analyze the structure of genetic diversity of *D. carota* and to develop a genetic map of carrot. Finally, information from the two DArT genotyping datasets was used to analyze genomic localization of regions differentiating the wild and the cultivated (Eastern and Western) carrot gene pools.

## Materials and methods

### Plant materials and DNA extraction

For the development of the carrot DArT microarray and diversity analysis, 159 DNA samples representing plants from 94 cultivated (*Daucus carota* L. subsp. *sativus* Hoffm.) and 65 wild (*D. carota* subsp.) accessions were used (Online Resource 1, Tables S1 and S2). Plants were grown in 3:1 sand and commercial humus mixture in 19-cm pots. Glasshouse conditions were optimized for carrot growth, i.e. 20–25 °C by day and 10–15 °C by night, and about 60 % relative humidity. Young leaves of individual plants were freeze-dried. After 105 days of vegetation, roots of the same plants were harvested and assessed for root color both at the root surface and cross-section, and for root shape.

The mapping population comprised 188 F2 [(Scarlet Nantes × Camberley) × (Turkish × 2566B)] individuals, where (Scarlet Nantes × Camberley) and (Turkish × 2566B) designate two inbred lines derived from crosses of the four parental stocks noted, and the F2 population was derived from self-pollinating a single F1 plant. The mapping population is henceforth referred to as 70349 F2. Ten randomly selected 70349 F2 plants were also used to develop a *D. carota* DArT microarray. DNA was extracted from either fresh or freeze-dried leaf tissue following the modified CTAB protocol (Briard et al. 2000). DNA concentration was adjusted to be in the range of 50–100 ng μl^−1^.

### Development of the DArT microarray

The first step in DArT genotyping comprises a reduction in genome complexity which involves digestion of genomic DNA with a set of selected restriction enzymes coupled with ligation of adapters specific to restriction site(s). Digestion and adapter ligation were performed simultaneously by adding 1 μl of genomic DNA solution to the total volume of 9 μl containing 2 units of *Pst*I, 2 units of *Taq*I, 80 units of T4 DNA ligase and 0.05 μM adapter (5′-CAC GAT GGA TCC AGT GCA-3′ annealed with 5′-CTG GAT CCA TCG TGC A-3′). Reactions were incubated at 37 °C for 2 h, followed by 2 h at 60 °C, as required by the enzyme combinations. 1 μl of digestion/ligation reaction product was used as a template for PCR amplification in a 50-μl reaction using DArT *Pst*I primer (5′- GAT GGA TCC AGT GCA G-3′) with the following cycling parameters: 94 °C for 1 min, followed by 30 cycles of 94 °C for 20 s, 58 °C for 40 s and 72 °C for 1 min, with a final extension at 72 °C for 7 min.

The genomic representations were pooled and cloned using the TOPO TA Cloning Kit (Invitrogen) following a protocol provided by the manufacturer. Transformant *E. coli* colonies were hand-picked and organized into twenty 384-well plates containing LB medium with 4.4 % glycerol and 100 μg ml^−1^ ampicillin, and grown at 37° for 18 h. Four plates comprising 1,536 clones were derived from genomic representations of the diversity collection, while 16 plates (6,144 clones) were derived from the 70349 F2 mapping population. Preparation of clones for spotting and printing of the DArT microarray was performed as described by Sansaloni et al. (2010). Two replicates per clone were spotted on each array. The operational array had 15,360 spots, comprising two randomly positioned spots for each one of the 7,680 clones.

Genomic representations of individuals representing 159 diversity accessions and 188 plants from the 70349 F2 mapping population were prepared as described above for library construction, to generate ‘targets’ for hybridization to the arrays. The targets were precipitated individually with isopropanol, washed with 70 % ethanol and air-dried at room temperature for 12 h. Targets were labeled in a 10-μl reaction with 2.5 nM of Cy3-dUTP or Cy5-dUTP (Amersham Bioscience), 2.5 units of Klenow exo-fragment of *E. coli* polymerase I (New England Biolabs) and 25 μM random decamers in 1× NEB Buffer 2 (New England Biolabs). The labelling reactions were incubated at 37 °C for 3 h. The DArT array is available to the public through Diversity Arrays Technology Pty. Ltd. (http://www.diversityarrays.com/).

### DArT microarray-based genotyping

Hybridization of targets, microarray scanning, image acquisition and analysis were essentially performed as described by Sansaloni et al. (2010). For selection of polymorphic markers, we used the following quality parameters: Call Rate (percentage of targets that could be scored as ‘0’ or ‘1’), *P* value (between-cluster variance in relative hybridization intensity as a percentage of the total variance), PIC (polymorphic information content), and OneRatio (proportion of ‘1’ in all reads).

### Analysis of the genetic diversity structure

Marker frequencies were used to calculate indices of marker information content and genetic diversity implemented in GenAlEx 6.4 (Peakall and Smouse 2006), viz. the mean number of alleles per locus, effective number of alleles (1/Σ *p*_*i*_^2^), number of rare (frequency < 0.05) and private alleles, Shannon’s index, PIC, and unbiased gene diversity UH_e_. Genetic structure was investigated using a Bayesian clustering approach without information on the accession origin and assuming the admixture model and correlated allele frequencies (STRUCTURE 2.2.3; Pritchard et al. 2000). Five independent simulations with a burn-in length of 10^4^ and a run length of 10^5^ were used for each number of clusters (*K*) set from 1 to 7. For the most likely number of genetic clusters, run parameters were increased by the factor of 10. That number of clusters was assumed from the peak value of distribution of the ad-hoc statistics Δ*K* as proposed by Evanno et al. (2005) using the online application Structure Harvester (Earl 2011). Divergence between identified clusters was summarized by partitioning genetic diversity using analysis of molecular variance (AMOVA). Genetic diversity was visualized in a scatter plot obtained after the principal coordinate analysis (PCoA) in GenAlEx.

### Construction of the genetic linkage map

JoinMap 4.0 software (Van Ooijen 2006) was used for mapping. Scores of all DArT markers were converted into genotype codes using the AC/BD system for dominant markers segregating in F2 populations. The linkage groups (LGs) were obtained at a LOD threshold value >3.0. We used regression mapping algorithm and the Kosambi mapping function to calculate distances between marker loci. The DArT markers were named with the prefix ‘crPt’ where ‘cr’ stands for carrot, ‘P’ for *Pst*I, and ‘t’ for *Taq*I, followed by numbers corresponding to their unique clone ID. Markers with more than 10 % of missing data were removed. Redundant markers were removed using the similarity loci function in JoinMap with a similarity threshold of 0.95. Each marker was tested against the expected segregation ratio using a Chi squared goodness of fit. The linkage map was first built with higher likelihood support for marker order following the second round of JoinMap. For the final map a third round of ordering of unmapped markers was performed using the fixed order function of markers as established at round two, allowing retention of the high likelihood support for markers as established at round two and providing the most likely position of all remaining informative markers.

LGs were anchored to chromosomes through physical co-localization of DArT sequences and currently available SNPs with known chromosome locations (Iorizzo et al. 2013b) on the assembled carrot contigs (Iorizzo et al. 2012). For each LG, a subset of DArT clones was randomly sequenced and the sequences were BLASTed against a draft assembly of the carrot genome (Iorizzo et al. 2012). For that purpose, DArT clones were PCR amplified and sequenced. Fragments were amplified in a 20-μl PCR reaction comprising 14 μl water, 2 μl 10× DNA polymerase buffer, 0.8 μl dNTPs (2.5 mM each), 1 μl of each 5 μM primer [pUC-M13Fw: CGCCAGGGTTTTCCCAGTCACGAC and pUC-M13Rv: TCACACAGGAAACAGCTATGAC (Integrated DNA Technologies, Coralville, IA, USA)], 0.2 μl *Taq* polymerase (MBI, Fermentas, USA) and 1 μl of clone culture. Amplification conditions were: initial denaturation at 94 °C for 2 min, followed by 35 cycles of 94 °C for 45 s, *T*_m_ (°C) for 60 s and 72 °C for 80 s, and a final step at 72 °C for 10 min. Amplification was confirmed by agarose gel electrophoresis of 5 μl of each PCR product, staining with ethidium bromide, and visualization under UV light. PCR products were then sequenced in a 5-μl reaction comprising 1.75 μl of water, 1 μl of 5 μM primer, 0.75 μl 5× BigDye^®^3.1 sequencing buffer, 0.5 μl of BigDye^®^3.1 ready reaction mix and 1 μl of PCR product, previously diluted 1:10 with water. Each insert was sequenced in both directions in separate reactions. Amplification conditions were: 25 cycles of 96 °C for 10 s and 58 °C for 2 min, and a final step at 72 °C for 5 min. The sequences were analyzed on an ABI 3730xl DNA Analyzer. Resulting sequences were trimmed of vector and assembled into consensus sequences using Sequencher software version 4.8 (GeneCodes Corporation, Ann Arbor, MI, USA). The consensus sequences were aligned using BLASTn to a local database containing a draft carrot genome assembly. DArT markers with sequences unambiguously matching anchored contigs were assigned to the corresponding chromosomes. After being assigned to chromosomes, LGs were labeled following the chromosome classification of Iovene et al. (2011). Sequences of the anchored DArT markers are reported in Online Resource 2.

### Search for signatures of domestication

We used two approaches to identify markers showing significant divergence from neutral loci resulting from selection in the course of carrot domestication. To identify outliers, we used a Bayesian approach, as implemented in BayeScan 2.1 (Foll and Gaggiotti 2008). To reveal loci under selection, we calculated posterior probabilities and accompanying log_10_(Bayes factors) for all markers polymorphic in the diversity collection. We used an initial burn-in of 5 × 10^4^ steps followed by 20 pilot runs of 5 × 10^3^ iterations, each with prior odds for neutral model set to 2 and a beta prior for *F*_IS_ = 0.342 (±0.048), as estimated from the previously reported SSR data for carrot (Baranski et al. 2012a). We reported on loci showing a posterior probability higher than 0.67, assuming that a posterior probability of 0.67–0.75 [log_10_(Bayes factor) > 0.3] should be treated cautiously, as weak evidence for selection, 0.76–0.90 [log_10_(Bayes factor) > 0.5] was substantial evidence, 0.91–0.99 [log_10_(Bayes factor) > 1] provided strong evidence, while 0.99 > [log_10_(Bayes factor)] > 2 was decisive. In the second approach, we used output from the analysis of the genetic diversity structure described above. A general linear model function implemented in TASSEL (Trait Analysis by aSSociation, Evaluation, and Linkage; Bradbury et al. 2007) was used to calculate marker *R* ^2^ values of association between the markers and identified gene pools. Marker frequencies were calculated for each gene pool separately. A pairwise test for significance of the differences between proportions was performed to identify markers discriminating gene pools and a two-tail significance level was reported for each comparison.

## Results

### Diversity analysis

For the collection of 159 cultivated and wild carrot accessions, 1,850 DArT markers were polymorphic. Of those, 950 markers appeared redundant based upon pairwise comparisons of marker distribution, and thus further analyses of diversity were performed with the selected 900 non-redundant markers which had the lowest amount of missing data (Online Resource 3, Fig. S1). Of the 900 DArT markers, only five were rare with a frequency below 0.05 while 29 markers were present in over 95 % of accessions (Table 1). Thus, setting the 0.05 frequency as the criterion, 866 markers were polymorphic. As DArT markers are dominant, two allelic states are distinguished but the effective number of alleles depending on the ratio of the signal presence and absence was *A* ^e^ = 1.667. The markers differed in their PIC value, which ranged from 0.031 to 0.375 with a mean value of 0.301 (standard error < 0.001). Seventy-nine percent of the markers were highly discriminating with PIC value above 0.25.

**Table 1:** Comparative parameters of genetic diversity for three clusters

Parameters	Total	Cluster 1: Eastern type	Cluster 2: Western type	Cluster 3: wild *D. carota*
Number of accessions (*N*)	159	20	74	65
Number of markers (*A* ^t^)	900	838	888	894
Number of polymorphic markers^a^	866	605	665	797
% of polymorphic markers	96.2	72.2	74.9	89.1
Number of fixed markers	0	159	55	12
Number of private markers (*A* ^p^)	–	0	1	8
Number of rare markers (*A* ^r^)^a^	5	19	28	31
Number of frequent markers (*A* ^f^)^a^	29	55	140	54
Mean number of effective alleles (*A* ^e^)	1.667 ± 0.009	1.467 ± 0.012	1.539 ± 0.011	1.611 ± 0.011
Mean polymorphic information content (PIC)	0.334 ± 0.0002	0.247 ± 0.006	0.259 ± 0.006	0.323 ± 0.005
Mean Shannon’s index (*I*)	0.561 ± 0.005	0.402 ± 0.009	0.477 ± 0.007	0.521 ± 0.006
Mean gene diversity (UHe) (Nei 1978)	0.382 ± 0.004	0.278 ± 0.007	0.320 ± 0.005	0.354 ± 0.005

Means are given with their standard errors

^a^Signal presence with the frequency *f*: 0.0 < *f* ≤ 0.05—rare marker, 0.95 ≤ *f* < 1.0—frequent marker, 0.05 < *f* < 0.95—polymorphic marker

**Fig. 1:**
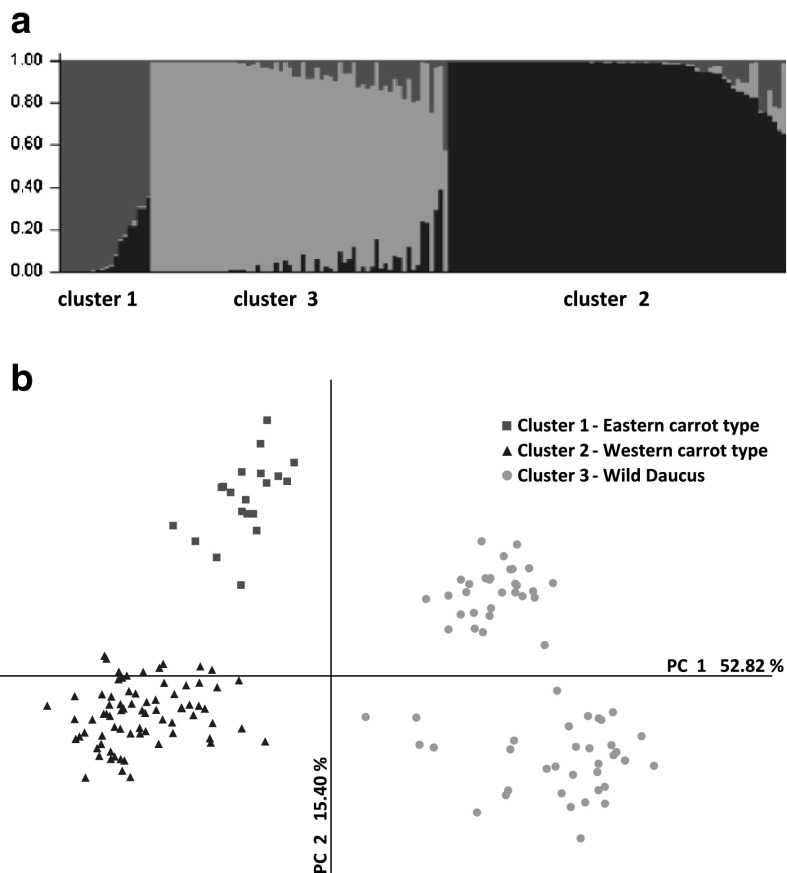
Structure of the genetic diversity of the 159 carrot accessions based on a Bayesian approach, assuming three clusters (**a**), and results of principal coordinate analysis (PCoA) of 159 carrot accessions based on 900 DArT markers (**b**)

The genetic structure of the collection was evaluated using a Bayesian approach. The Evanno Δ*K* statistic was the highest when assuming three clusters (Δ*K*_(3)_ = 161.1). Reduction of the number of clusters to two resulted in a two-fold lower Δ*K* value (Δ*K*_(2)_ = 79.1). Numbers of clusters higher than three were insignificant (Δ*K*_(*K*>3)_ = 0.2–0.6). Clusters 1, 2 and 3 comprised 20, 74 and 65 accessions, respectively (Fig. 1a). Most accessions (133 of 159) were assigned to one of the three clusters with a high probability ranging between 0.901 and 0.998. Only 18 accessions were assigned with the probability 0.701–0.900 and eight with the probability 0.581–0.700 (Online Resource 3, Fig. S2). PCoA was used to visualize diversity of the collection. Using the first three axes, 81.4 % of total variation could be explained, with the 1st, 2nd and 3rd axes explaining 52.8, 15.4 and 13.2 %, respectively. Using the first two axes, the accessions were arranged in three clearly separated groups that fully corresponded to the three clusters defined after a Bayesian simulation described above (Fig. 1b). The first axis differentiated clusters 1 and 2 from cluster 3 while the second axis mainly separated cluster 1 and cluster 2. AMOVA attributed 20 % of the total genetic diversity to variation between clusters.

Cluster 1 and cluster 2 included only cultivated carrots and cluster 3 included only wild relatives. Twenty accessions assigned to cluster 1 originated from continental Asia (14), Japan (2) and USA (4). Those assigned to cluster 2 were mainly of European origin (57) and the remaining accessions were from Japan (8), USA (5) and one accession each from Russia, Ethiopia, Australia and Brazil. The accessions belonging to clusters 1 and 2 differed in their root color. In cluster 1 only three accessions developed orange roots; the remaining accessions (17), including all those from USA and Japan, had yellow, red or purple roots. They developed mainly conical or narrow conical roots and only one accession (AS171) had cylindrical or tapered cylindrical roots. In contrast, the accessions in cluster 2 developed mainly orange roots (65 accessions); all accessions from USA and Japan had orange roots. White or yellow roots were produced by nine of 56 European accessions. Cylindrical or tapered cylindrical shape was characteristic for 27 of 74 accessions. Data on the origin of accessions supported by the morphological description indicate that cluster 1 comprised accessions of the Eastern carrot type while cluster 2 those of the Western type. Cluster 3 included only wild carrot subspecies of various origins. None of these accessions developed roots typical for edible carrot. Cluster 3 enclosed a distinct group of 30 accessions located in the first quarter of the PCoA plot and separated from the remaining accessions by the second axis. It contained all accessions obtained from the Greek gene bank collection, presumably collected in Greece, and additionally the accessions AS121 collected in Greece and AS138 in Spain. Most of the 900 DArT markers were shared among the three groups. No markers specific to cluster 2 (Eastern carrot) could be identified (Online Resource 3, Fig. S3).

Nei’s genetic distance between the two clusters of Eastern and Western gene pools (0.085) was smaller than between cultivated carrot and wild relatives: 0.141 for Eastern-wild and 0.127 for Western-wild. Gene diversity was the highest in the cluster of wild accessions (Table 1). It also contained eight private markers not present in any gene pool of cultivated carrot. Higher diversity was reflected also in a higher number of effective alleles, PIC and Shannon’s index values. The Eastern gene pool appeared to be less diverse than the Western gene pool, although diversity parameters for that group might be underestimated, as the number of accessions assigned to the Eastern gene pool was almost four times lower than the Western. Partitioning genetic diversity using AMOVA showed that 20 % of total variation was explained by divergence between the three gene pools and only 16 % when only cultivated carrots of Eastern and Western types were considered.

### Distribution of markers differentiating major gene pools

We searched for markers differentiating populations and showing signatures for selection in the course of domestication using a Bayesian approach implemented in BayeScan. The 900 DArT markers were ranked with regard to the highest posterior probability and the corresponding Bayes factor values (Online Resource 4). We set the cutoff at the posterior probability value of 0.67 and the corresponding log_10_(Bayes factor) value of 0.31, and identified 27 markers above the threshold, showing evidence for selection. There was a large decrease in these values for the remaining markers, the best not-qualified marker (ranked 28th) had a posterior probability of 0.60 and log_10_(Bayes factor) of 0.19. Four of the selected markers showed decisive evidence for directional selection, as they had posterior probabilities higher than 0.99 and Bayes factor higher than 100. Strong evidence for selection was observed for another six markers (posterior probability 0.91–0.99, Bayes factor > 10), while ten markers showed substantial evidence (posterior probability 0.76–0.90, Bayes factor > 3) (Table 2).

**Table 2:** DArT markers showing evidence for selection in the course of domestication, as revealed by BayeScan

Marker code	BayeScan results			*R* ^2^ value
	Posterior probability	Log_10_(Bayes factor)	Evidence for selection^a^	
crPt-895548	1.00	∞	Decisive	0.82
crPt-891401	1.00	2.49	Decisive	0.72
crPt-894083	0.99	2.02	Decisive	0.52
crPt-890483	0.99	2.01	Decisive	0.49
crPt-891238	0.97	1.48	Strong	0.39
crPt-893316	0.96	1.40	Strong	0.53
crPt-894175	0.95	1.32	Strong	0.52
crPt-884960	0.95	1.24	Strong	0.27
crPt-895381	0.92	1.08	Strong	0.51
crPt-894214	0.91	0.99	Strong	0.48
crPt-892544	0.90	0.94	Substantial	0.49
crPt-893913	0.86	0.78	Substantial	0.48
crPt-895800	0.84	0.71	Substantial	0.64
crPt-890436	0.83	0.69	Substantial	0.45
crPt-884180	0.82	0.65	Substantial	0.42
crPt-893685	0.79	0.57	Substantial	0.51
crPt-884542	0.79	0.57	Substantial	0.54
crPt-891552	0.77	0.52	Substantial	0.52
crPt-892661	0.77	0.52	Substantial	0.60
crPt-893851	0.76	0.50	Substantial	0.42
crPt-895780	0.75	0.50	Weak	0.50
crPt-890455	0.74	0.45	Weak	0.58
crPt-894182	0.72	0.42	Weak	0.28
crPt-884285	0.68	0.33	Weak	0.21
crPt-895746	0.68	0.33	Weak	0.42
crPt-894056	0.68	0.33	Weak	0.48
crPt-892106	0.67	0.32	Weak	0.27

^a^According to the Jeffreys’ scale of evidence for Bayes factors

**Fig. 2:**
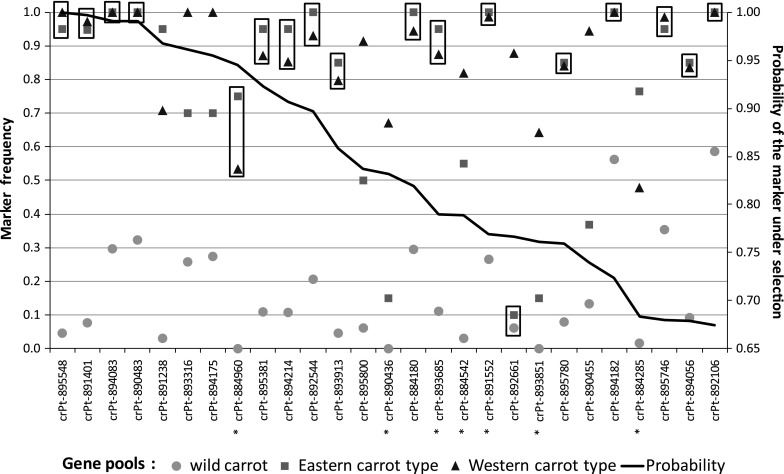
Frequency of markers with signatures for selection in the three carrot gene pools (wild, Eastern and Western). Marker frequencies not differentiating gene pools are *framed*. Markers with a shift in frequency towards the recessive ‘0’ allele (1—frequency) are labeled with *asterisks*

We further analyzed changes in allele frequencies among the three gene pools and identified 62 markers differentiating these groups of accessions with *R* ^2^ > 0.3 (Online Resource 4). All but four markers identified by BayeScan were included in that group. The highest *R* ^2^ value of 0.82 was observed for marker crPt-895548, followed by crPt-891401 (*R* ^2^ = 0.72) and crPt-895800 (*R* ^2^ = 0.62), while *R* ^2^ for the remaining markers was less than 0.6. For each marker we performed a pairwise test for the significance of difference between proportions. We grouped the 27 markers identified by BayeScan into three categories, those differentiating (I) wild/cultivated (Eastern and Western), (II) wild/Eastern/Western, and (III) wild and Eastern/Western, comprising 17, nine, and one marker, respectively (Fig. 2). Among those markers, we observed no markers with similar frequencies in the wild and the Western groups, which likely indicates a directional shift resulting from selection in the course of domestication. Only one marker, crPt-892661, showed a signature for selection in the Western gene pool (group III), while 17 were selected for upon domestication (group I). Thus, we speculate that the three categories represent (I) signatures for regions determining primary domestication traits, (II) signatures for regions determining traits differentiating Eastern and Western carrots, and (III) signatures for regions determining quality traits typical for Western carrots. Seven of the 27 markers showed a shift in frequency towards the recessive ‘0’ allele (Fig. 2). As DArT markers are dominant, only the recessive allele provides unambiguous information about the homozygosity of the particular variant and additional evidence for fixation of that variant.

### Genetic mapping

**Fig. 3:**
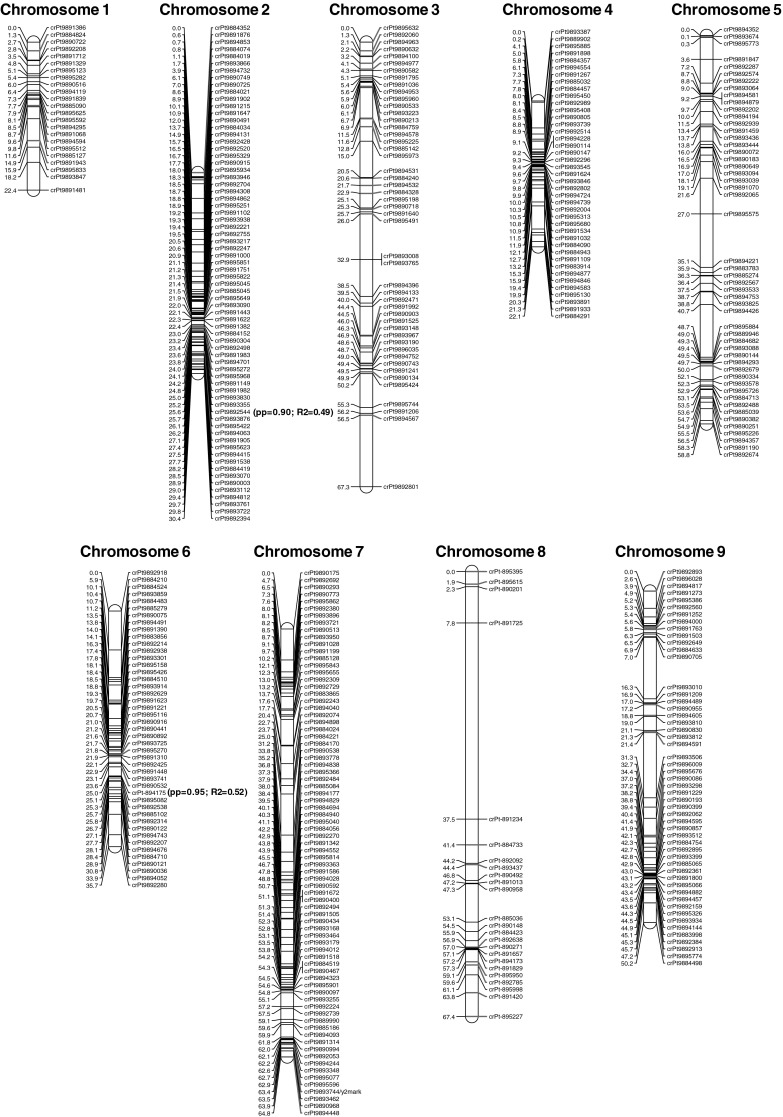
A genetic linkage map of the 70349 F2 carrot population constructed with 725 DArT markers. Markers labeled *red* were sequenced and used to physically attribute the linkage groups to chromosomes. A complete list of markers including redundant markers is given in Online Resource 5. Posterior probabilities and *R* ^2^ values for the two markers showing signatures for selection are shown in *green*. (Color figure online)

We identified a total of 1,208 DArT markers polymorphic in the 70349 F2 mapping population of carrot fulfilling the quality criteria, i.e. call rate higher than 80 % and *P* value higher than 67 %. The full set of markers was tested for segregation distortion (*χ* ^2^, *p* < 0.01) and redundancy, and 712 non-redundant DArT markers were retained for further analysis. Consistent with the carrot haploid chromosome number (*n* = 9), grouping analysis at LOD > 3.0 resulted in nine distinct groups of which eight contained from 33 to 118 markers, and the remaining group comprised only five markers. A genome sequence assembly anchored to a SNP-based linkage map of carrot (Iorizzo et al. 2013b) was used to associate groups to chromosomes, anchoring and orienting the linkage groups of the 70349 DArT map (see “Materials and methods”). In total, 56 markers distributed among the nine groups were anchored to carrot chromosomes (Fig. 3, markers highlighted in red). All anchored markers from each individual group physically mapped to a single chromosome, and all groups were unambiguously anchored to the nine carrot chromosomes. The linkage group with a low number of markers corresponded to chromosome 8. These results are consistent with recent mapping of SNP markers in another subpopulation of 70349 F2 where high segregation distortion of markers on chromosome 8 was found, resulting in the mapping of a very low number of non-distorted markers (Iorizzo et al. 2013b). To identify possible clusters of distorted markers on chromosome 8, the full DArT marker dataset including markers showing distorted segregation (*χ* ^2^, *p* < 0.001) was re-analyzed. At LOD > 3.0, 24 distorted markers grouped with the five non-distorted markers previously assigned to chromosome 8. Sequencing and analysis of all 24 DArT clones allowed the identification of seven markers physically linked to mapped markers on chromosome 8 (Iorizzo et al. 2013b), supporting the grouping analysis. To build the linkage map, these 24 distorted markers on chromosome 8 were included in the dataset of 712 non-distorted markers, resulting in 725 total markers. The final linkage map comprised 572 markers (Online Resource 1, Table S3) of which 431 (76 %) were unique, i.e. those markers demonstrating recombination with other DArT markers (Fig. 3). The number of markers per chromosome ranged from 28 (24 unique) for chromosome 8 to 108 (77 unique) for chromosome 7, with an average number of 63.6 (48.2 unique) markers. The total length of the map was 419.1 cM, with individual linkage groups ranging from 22.1 cM for chromosome 4 to 67.3 cM for chromosome 3. The average chromosome length was 46.6 cM and the average density of unique markers was 1.1 cM. Except for chromosome 8, where we observed a gap of 29.6 cM, only one map location (on chromosome 3) with gap greater than 10 cM was observed, indicating a relatively evenly distributed map coverage.

## Discussion

Diversity Arrays Technology has proved to be a robust source of dominant molecular markers used for analysis of genetic diversity and genetic mapping in plants (e.g. Wenzl et al. 2004; Yang et al. 2006; White et al. 2008; Bolibok-Bragoszewska et al. 2009). We developed and validated the first DArT genotyping platform for carrot comprising more than 7,000 DArT clones, of which ca. 1,000 produced high-quality non-redundant polymorphic signals in the diversity and/or mapping populations.

Here, we presented the first genetic map of carrot based on DArT markers. With 431 markers physically attributed to nine carrot chromosomes and an average distance between markers of 1.1 marker/cM, the map represents the densest map developed for carrot to date. Most markers on chromosome 8 showed high segregation distortion. Although genetic maps have been produced for several carrot populations (Just et al. 2007; Grzebelus et al. 2007; Cavagnaro et al. 2011; Alessandro et al. 2013; Yildiz et al. 2013), no severe segregation distortion has been reported for any particular carrot chromosome. Segregation distortion is influenced by many factors, including genetic factors, type of mapping population, their parents, and marker type (Liu et al. 2010). Consistent with our results, an extensive segregation distortion on chromosome 8 was observed while genotyping the population with SNP markers (Iorizzo et al. 2013b). These observations suggest that the phenomenon was not a result of the implemented marker technology. The presence of chromosomal structures causing meiotic drive, similar to that reported recently in maize (Kanizay et al. 2013), could be one of the possible explanations for that finding. However, to date no cytological evidence for the presence of knobs on carrot chromosomes was reported (Iovene et al. 2008, 2011; Nowicka et al. 2012). Other chromosome aberrations and genes that result in preferential segregation or survival of one chromosomal region relative to its homolog can also result in distorted segregation, and with the ongoing sequencing of the carrot genome, sequencing of distorted DArT makers on chromosome 8 will allow further investigation of biological factors influencing segregation distortion in the mapping population evaluated in this study.

Despite the lower discriminatory power of dominant markers for genetic diversity analysis, DArT markers allowed clear differentiation of three distinct carrot gene pools: wild, Eastern-cultivated and Western-cultivated. Most of the accessions investigated were unambiguously attributed to one of these groups. Comparison of the DArT results and the results of a previous diversity analysis with 30 SSR markers (Baranski et al. 2012a) for a subset comprising 88 accessions investigated in both studies revealed that both marker systems allowed the identification of two distinct gene pools of cultivated carrot. In this comparison, DArT markers had much higher discriminatory power, due to the fact that they were generated in a high-throughput procedure resulting in hundreds of informative markers. This was reflected by almost two-fold higher divergence between gene pools using DArT markers (*F*_ST_ = 0.16; *p* < 0.01) than SSR markers (*F*_ST_ = 0.097) and confirmed by AMOVA attributing 16.0 and 8.8 % of total genetic variation between gene pools for DArT and SSR markers, respectively. In consequence, all accessions investigated could have been assigned to either the Eastern or Western gene pool using DArT markers, while using SSR markers 14 % of accessions remained unassigned and the assignment of several other accessions was ambiguous (Online Resource 1, Table S4). Seventy-six of 78 accessions were classified to the same gene pools using these two different molecular marker systems. Only two accessions originally classified to the Eastern gene pool using SSR markers were placed in the Western gene pool following analysis with DArT markers. Classification of Long Red (AS87) was different using the two systems; with SSR markers it was classified to the Eastern gene pool with the probability *p* = 0.93 and with DArT markers it was classified to the Western gene pool with *p* = 0.99. Classification of Yellowstone (AS02) to the Eastern gene pool using SSR markers might have been ambiguous (*p* = 0.67) while DArT markers provide clear indications for its similarity to Western carrots (*p* = 0.99). Ten accessions not assigned to any gene pool using SSR markers were classified to either Eastern (Persia AS12) or Western (9 accessions) gene pools with high probabilities.

Using 4,000 SNPs, Iorizzo et al. (2013a) also differentiated three major gene pools and showed that wild carrots originating from Central Asia were the most closely related to the Eastern cultivated carrots, supporting the hypothesis on the Central Asian origin of domesticated carrot. Our results indicated a directional shift in the frequency of some DArT markers, likely resulting from the selection pressure imposed in the course of domestication and further improvement, constituting genomic signatures for selection in the carrot genome. The extent of that shift in allele frequency placed Eastern carrots in the intermediate position between the wild and the Western cultivated carrots and corroborated the hypothesis that orange Western carrots were selected from the more primitive Asian gene pools proposed by Banga (1957) and confirmed by the recent SNP study (Iorizzo et al. 2013a). Moreover, we were able to divide the markers showing signatures for selection into three categories, i.e. those that were selected primarily in the cultivated carrots, representing primary domestication events, those under continuous selection from wild to Eastern to Western, and those differentiating Western from both Eastern and wild, representing secondary domestication events, likely related to traits differentiating Eastern and Western cultivated carrots.

Only two of the 27 DArT markers showing strong signatures for selection segregated in the mapping population and we were able to determine their position on the genetic map. This could have been expected, as regions governing domestication-related traits were mostly invariant in the cultivated carrot, and hence also in the mapping population. The two markers mapped to chromosomes 2 and 6 (Fig. 3), providing evidence that certain regions on these chromosomes were selected for in the cultivated carrots. The marker crPt-892544, localized on chromosome 2, showed a shift in the frequency from wild to cultivated and did not differentiate Eastern and Western cultivated. Interestingly, chromosome 2 was previously shown to be carrying the *Vrn1* gene responsible for early flowering habit (Alessandro et al. 2013), which was an apparent target for selection in the course of carrot domestication. Thus, it might represent a signature for selection towards biennial growth habit—one of the hallmark primary domestication traits in carrot. In contrast, the marker ctPt-894175, mapped to chromosome 6, showed a directional shift from low frequency in wild to increased frequency in Eastern to high in Western gene pools. Further analysis of the signatures identified for selection would likely provide a valuable insight into the nature of genomic regions subject to artificial selection by early farmers and modern breeders.

## Electronic supplementary material

Below is the link to the electronic supplementary material.

Supplementary material 1 (PDF 215 kb)

Supplementary material 2 (TXT 24 kb)

Supplementary material 3 (PDF 140 kb)

Supplementary material 4 (XLS 286 kb)

Supplementary material 5 (XLS 64 kb)
