# Supplementary material for: Diversity, genetic mapping, and signatures of domestication in the carrot (Daucus carota L.) genome, as revealed by Diversity Arrays Technology (DArT) markers
Source: Mol Breed. 2013 Oct 26;33(3):625–37. doi: 10.1007/s11032-013-9979-9 (PMC3918115; doi:10.1007/s11032-013-9979-9)
Supplement: Supplementary file 1 — Supplementary material 1 (PDF 215 kb) [file 11032_2013_9979_MOESM1_ESM.pdf]

Table S1. List of cultivated carrot accessions used in the diversity analysis

Ref. No.	Source / No.	Name	Donor country[a]	Origin	Status[b]	Root color[c]	SSR gene pool[d]	DArT gene pool[e]
AS 02	Bejo	Yellowstone	NLD	Europe	OP	Y	E	W
AS 04	Seminis	Nutrired	USA	USA	OP	R	-	E
AS 09	Seminis	Anthonina	USA	USA	OP	P	E	E
AS 11	INH / 126	Blanche 1/2 longue des vosges	FRA	Europe	OP	W	na	W
AS 12	WGRU / 3931	Persia No. 242	IRN	Asia	LR	(Y)W	na	E
AS 13	WGRU / 3921	Gelbe Rheinische	DEU	Europe	OP	Y	W	W
AS 14	DAU / 315	BL-JKI-1	TUR	Asia	BP	O	E	E
AS 15	WGRU / 11201	Afghan Purple	USA	USA	LR	P	E	E
AS 16	DAU / 437	Senta	DEU	Europe	OP	O	na	W
AS 18	WGRU / 5779	Nagykallo	HUN	Europe	OP	O	W	W
AS 19	WGRU / 11718	Himuro Fuyugosi Gosun No.2	JPN	JPN	OP	O	W	W
AS 20	POLAN	Nantejska Polana	POL	Europe	OP	O	W	W
AS 21	MKS	Hakata Kintoki	JPN	JPN	OP	R	E	E
AS 22	WGRU / 6755	Pusa Kesar	IND	Asia	LR	O	E	E
AS 23	WGRU / 6754	Panipat Special	IND	Asia	LR	(R)O	E	E
AS 24	WGRU / 13238	Syrian Purple	TUR	Asia	LR	P	E	E
AS 25	Wild	BL-JKI-2	TUR	Asia	BP	P	E	E
AS 27	MKS	China Yellow	CHN	Asia	OP	Y	E	E
AS 28	WGRU / 6752	Shahpur Special	IND	Asia	LR	R	E	E
AS 29	MKS	Kokubu Senko Oonaga	JPN	JPN	OP	O	na	W
AS 30	MKS	Sapporo Futo	JPN	JPN	OP	O	W	W
AS 31	MKS	Shima Ninjin	JPN	JPN	OP	Y	E	E
AS 32	MKS	Hekinan Senko 5sun	JPN	JPN	OP	O	W	W
AS 33	Commercial	Amsterdam 3	POL	Europe	OP	O	W	W
AS 34	BVRC	BL-H119	CHN	Asia	BP	(R)O	-	E
AS 35	WGRU / 8720	White Belgian	GBR	Europe	OP	W	W	W
AS 36	Commercial	Lobbericher	DEU	Europe	OP	Y	na	W
AS 37	WGRU / 10146	Gajar (10146)	PAK	Asia	LR	R	E	E
AS 38	WGRU / 13403	Mestnaya (13403)	RUS	Asia	LR	W	na	W
AS 39	JKI	BL-JKI-7	DEU	Asia	BP	P	E	E
AS 40	JKI	BL-JKI-6	DEU	Asia	BP	(R)Y	W	W
AS 41	WGRU / 10627	Lozin147	CSK	Europe	OP	O	W	W
AS 42	WGRU / 7125	Delta a Cuoro Rosso	ITA	Europe	OP	O	W	W
AS 43	USDA	HCM	USA	USA	OP	O	W	W
AS 44	WGRU / 8390	Western Red	AUS	Australia	OP	O	W	W
AS 45	WGRU / 3842	Norfolk Giant	GBR	Europe	OP	O	W	W
AS 46	WGRU / 9808	Kuettiger (9808)	CHE	Europe	OP	W	na	W
AS 47	DAU / 333	Yellow Belgian	NLD	Europe	OP	Y	W	W
AS 48	WGRU / 10480	Bitolski	YUG	Europe	OP	O	W	W
AS 49	WGRU / 10197	Gajar (10197)	IND	Asia	LR	O	E	E
AS 50	WGRU / 13405	Mestnaya (13405)	RUS	Asia	LR	(P)Y	E	E
AS 51	WGRU / 3982	Red Elephant	GBR	Europe	OP	O	W	W
AS 52	WGRU / 3849	Victa	GBR	Europe	OP	O	W	W
AS 53	WGRU / 4001	Niiza Etton Gosun	JAP	JPN	OP	O	W	W
AS 54	Bejo	Nevis	NLD	Europe	OP	O	W	W

AS 55	Sperli	Rotin	DEU	Europe	OP	O	W	W
AS 56	Bejo	Vita Longa	NLD	Europe	OP	O	W	W
AS 57	NGB / 1852	Nantes Empire	DNK	Europe	OP	O	W	W
AS 58	NGB / 548	Touchon Format	DNK	Europe	OP	O	W	W
AS 59	NGB / 1863	Amsterdamer Master	DNK	Europe	OP	O	W	W
AS 60	DAU / 341	Lange Rote Stumpfe ohne Herz 1	DEU	Europe	OP	O	W	W
AS 61	NGB / 13970	Nana W 561	SWE	Europe	OP	O	W	W
AS 62	WGRU / 4002	Shinsuu Senkou Oonaga	JPN	JPN	OP	O	W	W
AS 63	WGRU / 7801	Benifuku Fuyngosi 5 Sun	JPN	JPN	OP	O	W	W
AS 64	WGRU / 10626	Stratova	CSK	Europe	OP	O	W	W
AS 65	DAU / 326	Nantes Half Long	m	Europe	OP	O	W	W
AS 66	WGRU / 3971	Champion Scarlet Horn	GBR	Europe	OP	O	W	W
AS 67	WGRU / 3844	Viking	GBR	Europe	OP	O	W	W
AS 68	WGRU / 11157	Berlicum Normaal	NLD	Europe	OP	O	W	W
AS 69	WGRU / 6519	Purple Stem Selektion	FRA	Europe	OP	Y	W	W
AS 70	DAU / 424	Danver's Red Core	m	USA	OP	O	W	W
AS 71	NGB / 1855	Nantes Duke	DNK	Europe	OP	O	W	W
AS 72	WGRU / 3835	Red Giant	GBR	Europe	OP	O	W	W
AS 73	Vilmorin	Bolero	FRA	Europe	F1	O	-	W
AS 74	Vilmorin	Texto	FRA	Europe	F1	O	W	W
AS 75	Vilmorin	Presto	FRA	Europe	OP	O	W	W
AS 76	USDA	Beta III	USA	USA	OP	O	W	W
AS 77	JKI	Pariser Markt	DEU	Europe	OP	O	W	W
AS 78	JKI	Vitaminaja	RUS	Europe	OP	O	W	W
AS 79	WGRU / 8863	Cyrano	NLD	Europe	OP	O	W	W
AS 80	DAU / 460	Gold Pak	m	USA	OP	O	W	W
AS 81	Bejo	Rainbow	NLD	Europe	F1	O	W	W
AS 82	Bejo	Mello Yello	NLD	Europe	F1	Y	na	W
AS 83	WGRU / 6788	St. Valery	POL	Europe	OP	O	W	W
AS 84	WGRU / 3955	Sytan	FRA	Europe	OP	O	W	W
AS 85	NGB / 551	Nantes Liva	DNK	Europe	OP	O	W	W
AS 86	NGB / 1849	Flakkeer Regina	DNK	Europe	OP	O	W	W
AS 87	WGRU / 10246	Long Red	ETH	Africa	LR	Y	E	W
AS 88	WGRU / 9325	Winterperfection	NLD	Europe	OP	O	W	W
AS 89	WGRU / 7884	Stupicka k Rychleni	CSK	Europe	OP	O	W	W
AS 90	WGRU / 7265	Short n'Sweet	USA	USA	OP	O	na	W
AS 91	WGRU / 7126	Tropical	BRA	S-America	OP	O	na	W
AS 92	WGRU / 6688	Moskovskaja Zimniaja	RUS	Europe	OP	O	W	W
AS 93	WGRU / 6183	Nyiregyhaza	HUN	Europe	OP	O	-	W
AS 94	WGRU / 11715	Yamanouchi Ishyaku Senko	JPN	JPN	OP	O	W	W
AS 95	NGB / 2399	London Torve, B Tagenshus III	DNK	Europe	OP	O	W	W
AS 96	WGRU / 5593	Berlicumer Bercoro	NLD	Europe	OP	O	W	W
AS 97	NGB / 1857	Nantes Palisade	DNK	Europe	OP	O	W	W
AS 98	WGRU / 6085	Berlikum Perfecta	ITA	Europe	OP	O	W	W
AS 99	WGRU / 6026	Beacon	GBR	Europe	OP	O	W	W
AS 100	NGB / 13955	Regulus Imperial	SWE	Europe	OP	O	W	W
Res2	Bejo	Romosa	NLD	Europe	OP	O	W	W
AS 124	DAU / 261	*D.c.carota* [f]	IRQ	Asia	m	O	-	E
AS 171	JKI	*D.c.carota*[f]	USA	USA	m	O	-	E

[a] Country of donor; m – missing data

[b] OP – open pollinated cultivar, F1 – hybrid, BL – breeding population, LR – landrace; m – missing data

[c] Color of root phloem, in brackets color of root surface if different than phloem color; O – orange, Y – yellow, W – white, R – red, P - purple

[d] Assignment to the Eastern (E) and the Western (W) gene pools was done previously based on polymorphisms of SSR loci (Baranski et al. 2012a); na – not assigned

[e] Assignment to the Eastern (E) and the Western (W) gene pools based on the results presented in this paper

[f] Originally described as *D. c. carota*, but the plants developed orange storage root typical for cultivated carrot

Bejo – Bejo Zaden B.V., Warmenhuizen, The Netherlands; BVRC – Beijing Vegetable Research Center, Beijing, China; DAU – Leibniz Institute of Plant Genetics and Crop Plant Research, IPK-Gatersleben, Germany; INH – Institut National d'Horticulture et de Paysage, Angers, France; JKI – Julius Kuehn Institute, Quedlinburg, Germany; MKS – Mikado Kyowa Seed Co. Ltd., Chosei, Japan; NGB – Nordic Genetic Resource Center, Alnarp, Sweden; POLAN – Krakowska Hodowla i Nasiennictwo Ogrodnicze POLAN Ltd., Krakow, Poland; Seminis – Seminis Vegetable Seeds, Inc., Saint Louis, USA; Sperli – Saatzucht Carl Sperling & Co. GmbH, Lüneburg, Germany; USDA – USDA-ARS, University of Wisconsin, Department of Horticulture, Wisconsin, USA; Vilmorin – Vilmorin & Cie Co., La Ménitré, France; WGRU – Warwick Genetic Resources Unit, Warwick University, Wellesbourne, Great Britain; Wild – Rudolf Wild GmbH & Co. KG, Berlin, Germany; commercial – seeds from shop

Table S2. List of wild carrot accessions used in diversity analysis

Ref. No.	Source[a] / No.	*Species*	Donor country
AS 111	USDA / 289-1	*D.c. hispidus*	ESP
AS 112	USDA / 274-1	*D.c. maritimus*	ESP
AS 113	USDA / PI295862	*D. c. maximus*	ESP
AS 114	USDA / 288-1	*D.c. maximus*	unknown
AS 120	DAU / 216	*D.c. carota*	AUT
AS 121	DAU / 218	*D.c.maximus*	GRC
AS 122	DAU / 252	*D.c.maximus*	ESP
AS 123	DAU / 257	*D.c.maximus*	SVN
AS 125	DAU / 307	*D.c.carota*	DEU
AS 126	DAU / 377	*D.c.carota*	DEU
AS 127	DAU / 383	*D.c.carota*	ITA
AS 129	DAU / 429	*D.c.carota*	DEU
AS 131	DAU / 217	*D.c.major*	FRA
AS 132	DAU / 240	*D.c.major*	FRA
AS 137	WGRU / 7191	*D.c.carota*	ESP
AS 138	WGRU / 7192	*D.c. carota*	ESP
AS 139	WGRU / 9212	*D.c.gummifer*	FRA
AS 141	WGRU / 9226	*D.c.carota*	CHE
AS 142	WGRU / 9202	*D.c.carota*	FRA
AS 143	WGRU / 9217	*D.c.maritimus*	ESP
AS 144	WGRU / 9218	*D.c.maximus*	ESP
AS 145	WGRU / 9203	*D.c.gummifer*	FRA
AS 147	JKI	*D.c.gadecai*	ESP
AS 148	JKI	*D.c. gummifer*	ESP
AS 149	JKI	*D.c. commutatus*	ESP
AS 150	JKI	*D.c. hispanicus*	ESP
AS 151	JKI	*D.c. drepanensis*	ESP
AS 152	JKI	*D.c. azoricus*	ESP
AS 153	JKI	*D.c.hispidifolius*	South America
AS 154	JKI	*D.c.libanotifolia*	ISR
AS 165	JKI	*D.c. carota*	USA
AS 166	JKI	*D.c. carota*	USA
AS 167	JKI	*D.c. carota*	USA
AS 168	JKI	*D.c. carota*	USA
AS 172	JKI	*D.c. carota*	USA
AS 173	JKI	*D.c. carota*	FRA
AS 174	JKI	*D.c. carota*	FRA
AS 182	GRC / 11001	*D.c.carota*	GRC
AS 184	GRC / 11007	*D.c.maximus*	GRC
AS 185	GRC / 11009	*Daucus carota*	GRC
AS 187	GRC / 11014	*Daucus carota*	GRC
AS 188	GRC / 11022	*D.c.maxima*	GRC
AS 189	GRC / 11024	*Daucus carota*	GRC
AS 190	GRC / 11027	*Daucus carota*	GRC
AS 193	GRC / 11030	*Daucus carota*	GRC
AS 194	GRC / 11031	*Daucus carota*	GRC
AS 195	GRC / 11034	*Daucus carota*	GRC

AS 196	GRC / 11035	*Daucus carota*	GRC
AS 197	GRC / 11036	*Daucus carota*	GRC
AS 198	GRC / 11037	*Daucus carota*	GRC
AS 199	GRC / 11039	*Daucus carota*	GRC
AS 200	GRC / 11041	*Daucus carota*	GRC
AS 201	GRC / 11043	*Daucus carota*	GRC
AS 202	GRC / 11048	*Daucus carota*	GRC
AS 203	GRC / 11050	*Daucus carota*	GRC
AS 204	GRC / 11055	*Daucus carota*	GRC
AS 205	GRC / 11057	*Daucus carota*	GRC
AS 207	GRC / 11064	*Daucus carota*	GRC
AS 208	GRC / 11066	*Daucus carota*	GRC
AS 209	GRC / 11067	*Daucus carota*	GRC
AS 210	GRC / 11071	*Daucus carota*	GRC
AS 211	GRC / 11073	*Daucus carota*	GRC
AS 212	GRC / 11075	*Daucus carota*	GRC
AS 213	GRC / 11076	*Daucus carota*	GRC
AS 214	GRC / 11077	*Daucus carota*	GRC

[a] GRC – Greek Gene Bank, Agricultural Research Centre of Makedonia and Thraki, Thermi – Thessaloniki, Greece;

for the remaining source abbreviations see Table 1

Table S3. Summary of mapping results in the 70349 F2 population of carrot

	Chromosomes									Average	Total
	**1**	**2**	**3**	**4**	**5**	**6**	**7**	**8**	**9**		
**Number of loci in group**	33	102	65	108	79	118	108	32	80	80.6	725
**Number of mapped loci**	30	93	60	56	78	55	108	28	64	63.6	572
**Number of mapped unique loci**	23	70	48	41	51	45	77	24	52	47.9	431
**Map length (cM)**	22.4	30.4	67.3	22.1	58.8	35.7	64.8	67.4	50.2	46.57	419.1
**Map density (markers/cM)***	0.97	0.43	1.40	0.54	1.15	0.79	0.84	2.81	0.97	1.10	0.97

*Based on unique markers

Table S4. Number of the same accessions classified to gene pools using either SSR or DArT markers

Gene pool	Number of accessions classified to gene pools using SSR markers*	Number of the same accessions classified to gene pools using DArT markers	
		Eastern gene pool	Western gene pool
Eastern	17	15	2
Western	61	0	61
not assigned	10	1	9

* Results from Baranski et al. 2012a
