# Supplementary material for: Diversity, genetic mapping, and signatures of domestication in the carrot (Daucus carota L.) genome, as revealed by Diversity Arrays Technology (DArT) markers
Source: Mol Breed. 2013 Oct 26;33(3):625–37. doi: 10.1007/s11032-013-9979-9 (PMC3918115; doi:10.1007/s11032-013-9979-9)

Figure S1. Number of loci (left) and accessions (right) with missing DArT data. Lines show cumulative percentage.

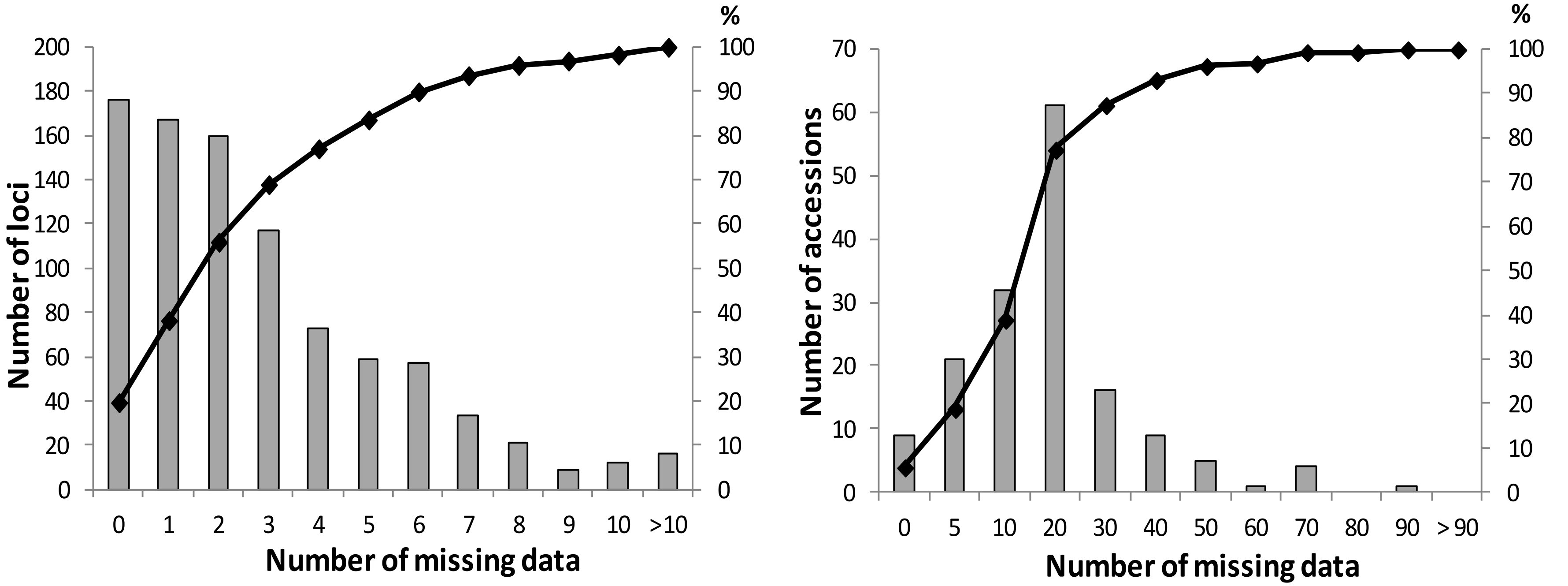

Figure S2. Cumulative assignment of carrot accessions to clusters with increasing probability of clustering assuming K=3, based on a Bayesian approach.

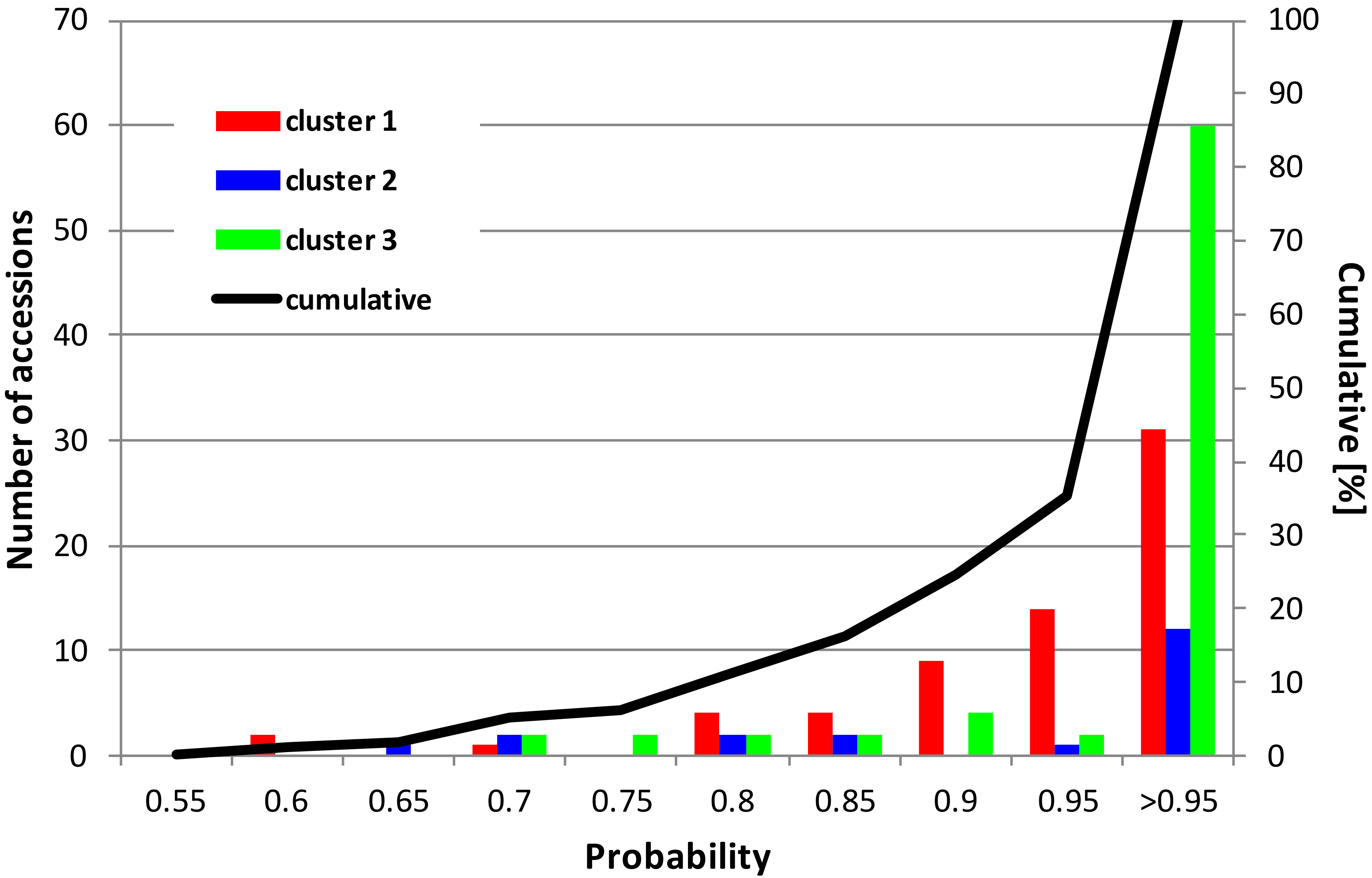

Figure S3. Venn diagram showing distribution of the 900 DArT markers in the three carrot gene pools – Eastern cultivated (cluster 1), Western cultivated (cluster 2), and wild (cluster 3), represented by purple, orange, and green circles, respectively.

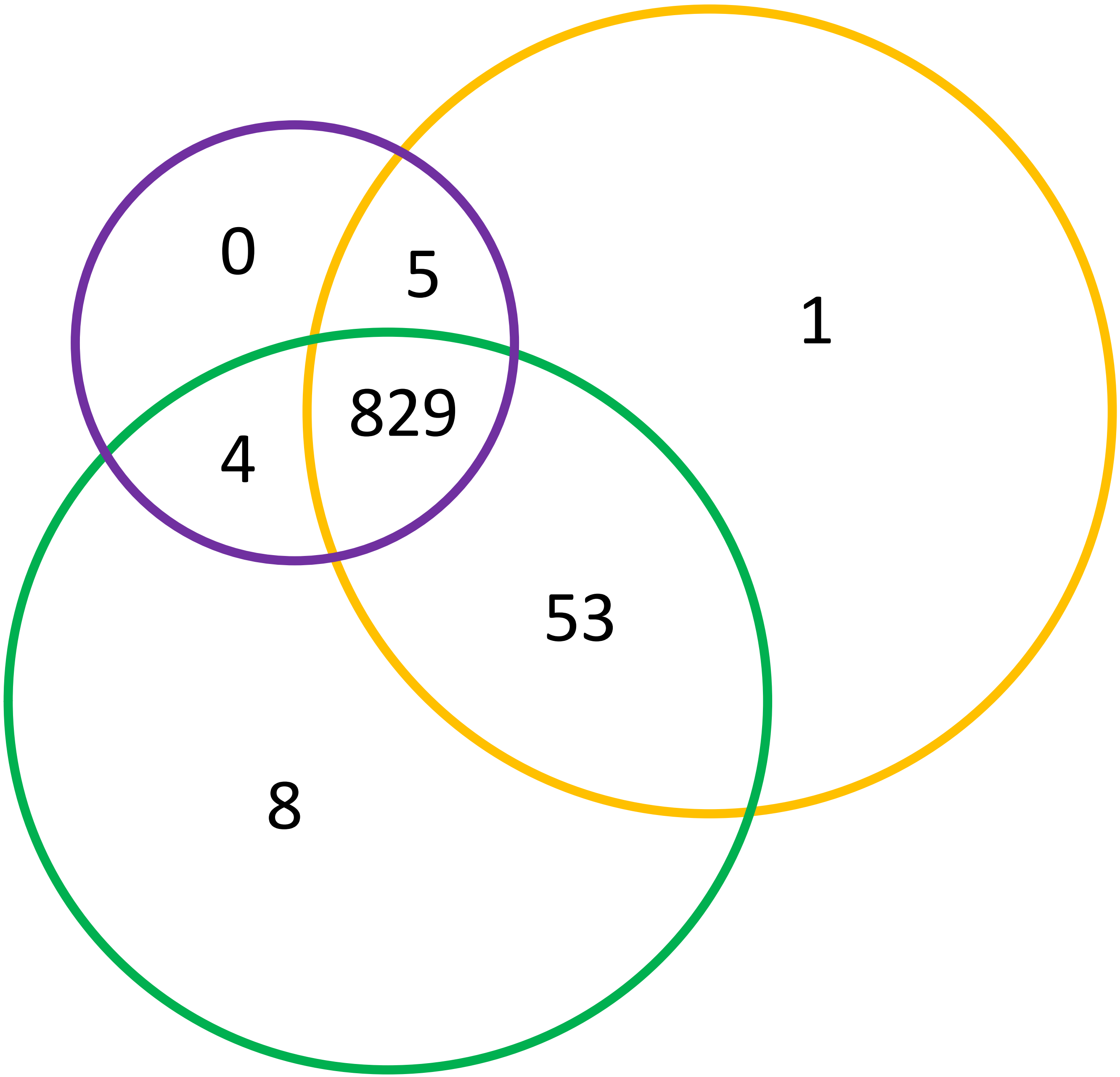

Supplement: Supplementary file 3 — Supplementary material 3 (PDF 140 kb) [file 11032_2013_9979_MOESM3_ESM.pdf]
